# Supplementary figures and images for: Effects of short-term isolation on social behaviors in prairie voles
Source: PLoS One. 2024 Nov 11;19(11):e0313172. doi: 10.1371/journal.pone.0313172 (PMC11554233; doi:10.1371/journal.pone.0313172)

A

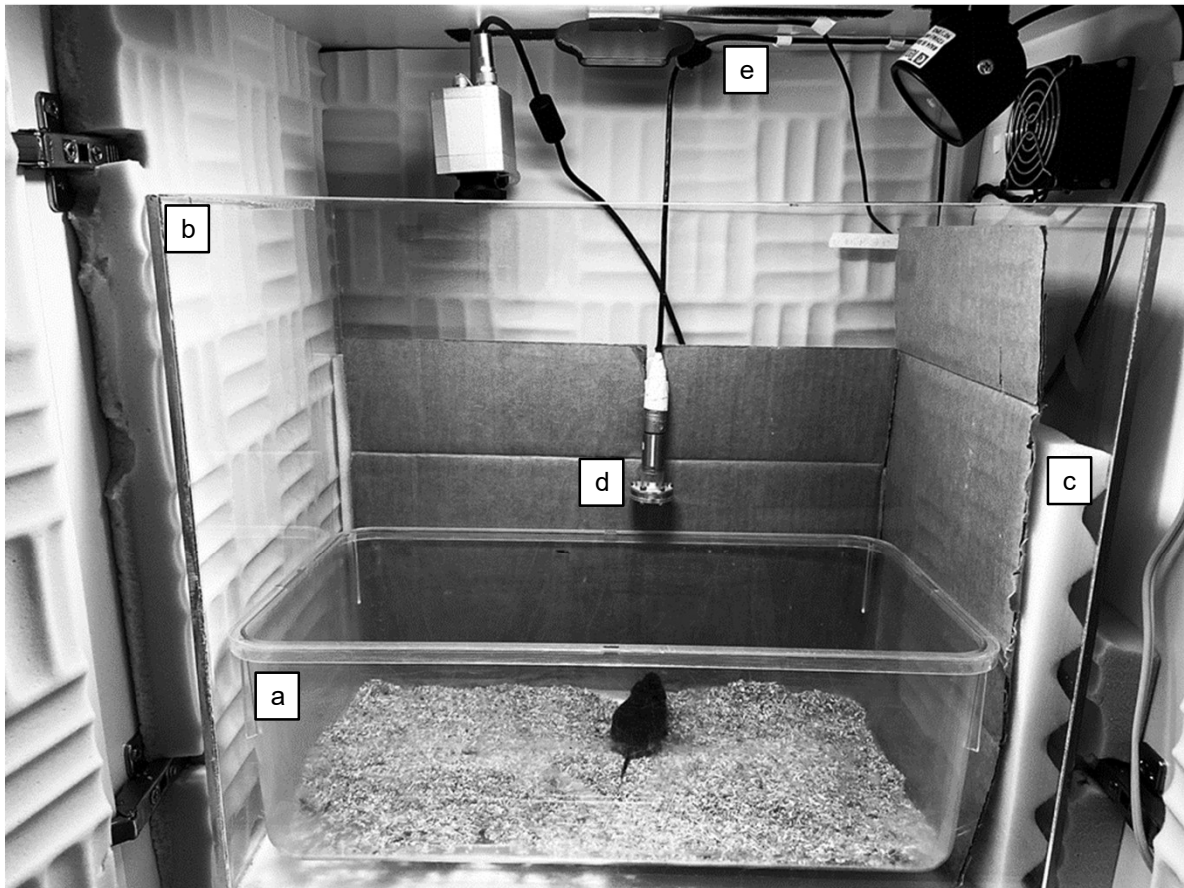

B

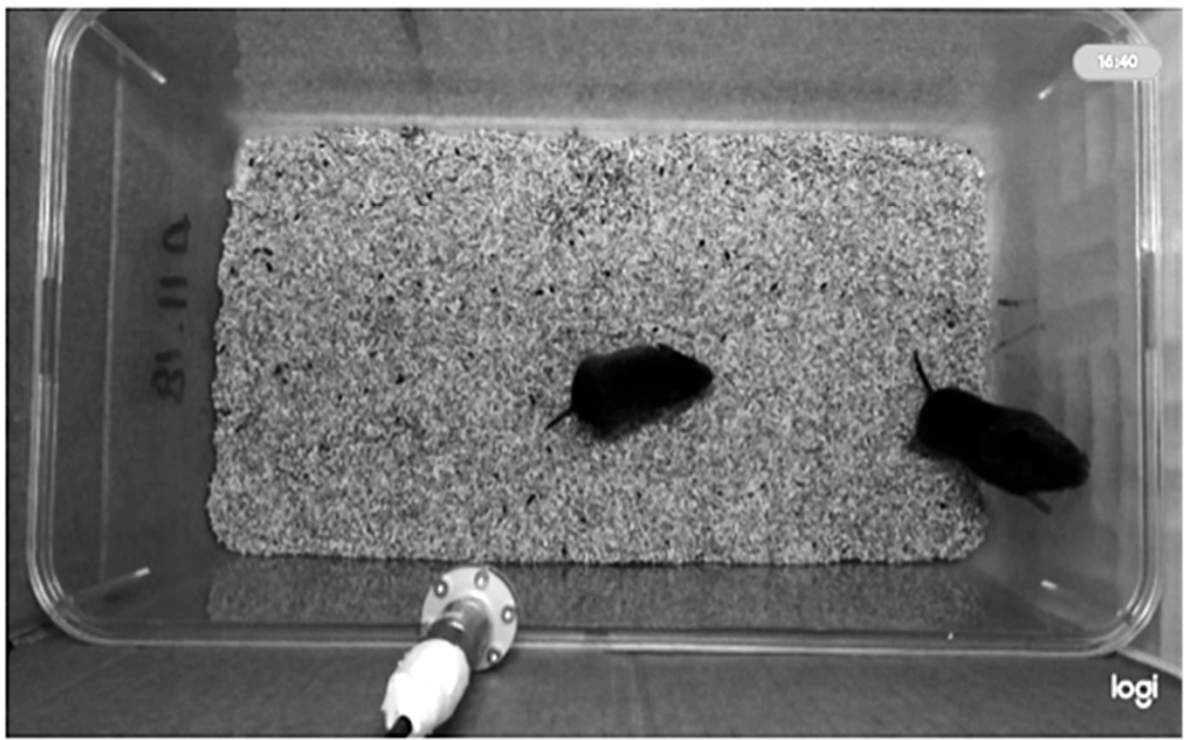

S1 Fig

Supplement: S1 Fig — (A) The home cage of the resident vole (a) was placed inside a plexiglass sleeve (b), with a small amount of foam padding (c) placed to fill any gaps between the edge of the home cage and the wall of the sleeve. The chamber was equipped with an ultrasonic microphone (d) and a webcam (e). Please note that although only the resident vole is present in this image, both a resident and visitor vole were placed in the chamber for each social interaction test. (B) View of the behavioral chamber from the overhead webcam. (PDF) [file pone.0313172.s001.pdf]

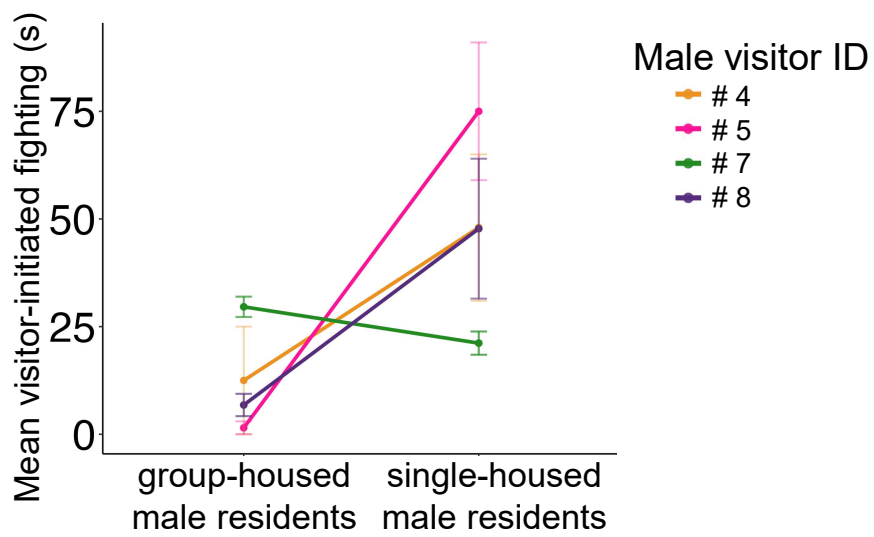

S2 Fig

Supplement: S2 Fig — Mean time (in seconds) that male visitors engaged in visitor-initiated aggressive behavior when interacting with group-housed vs. single-housed male residents. Lines and data points are color-coded by the identity of the male visitor. Data points show mean values, and error bars indicate standard errors. Visitor males #4 and #5 were each used in n = 2 trials with group-housed (GH) residents and n = 2 trials with single-housed (SH) residents. Visitor male #7 was used in n = 5 trials with GH residents and n = 6 trials with SH residents. Visitor male #8 was used in n = 5 trials with GH residents and n = 4 trials with SH residents. (PDF) [file pone.0313172.s002.pdf]

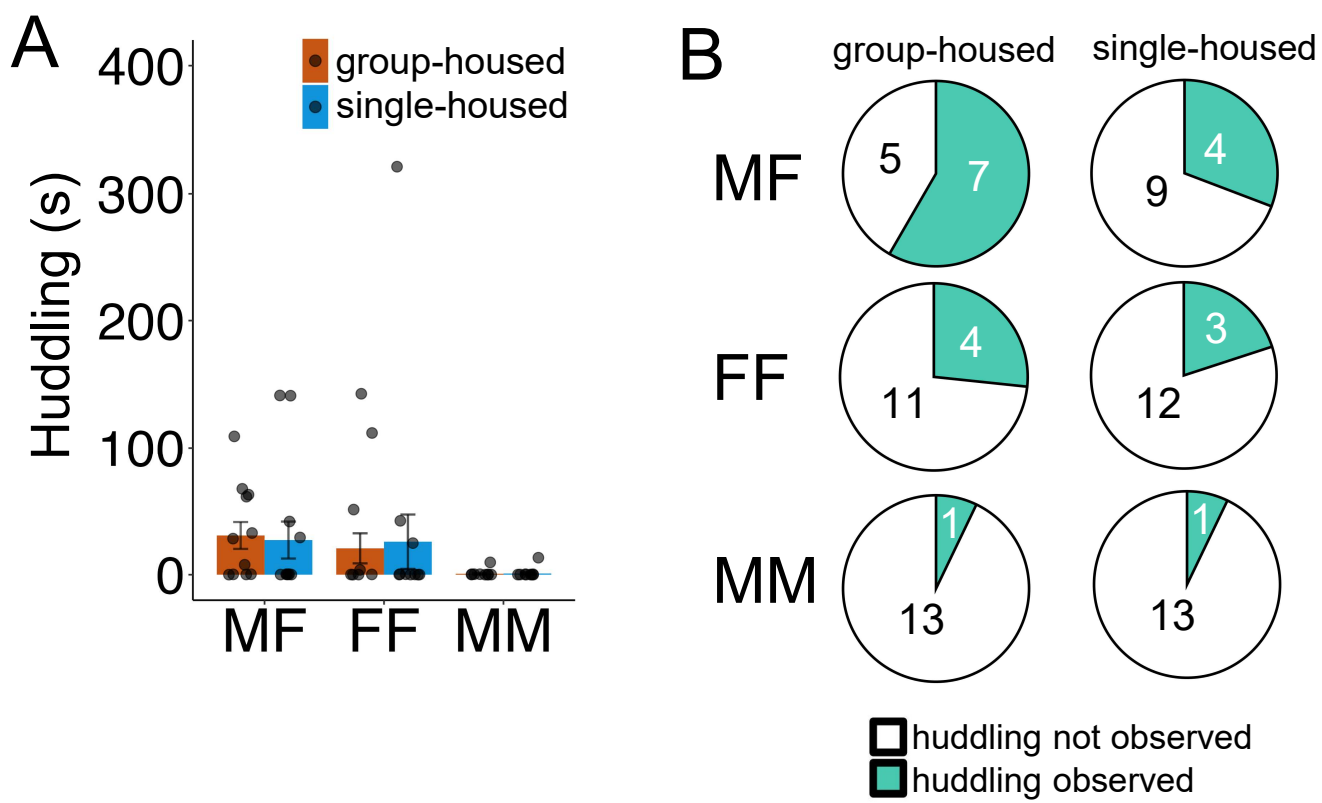

S3 Fig

Supplement: S3 Fig — (A) Time (in seconds) that pairs of voles spent engaged in huddling during social interaction trials is shown. Orange, trials with group-housed residents; blue, trials with single-housed residents. Bars indicate mean values, and error bars indicate standard errors. (B) Pie charts showing the number of pairs engaged in huddling in MF (top), FF (middle), and MM (bottom) social interactions, shown separately for pairs that included group-housed residents vs. single-housed residents. White shading indicates the proportion of trials in which pairs of voles did not huddle, and teal shading indicates the proportion of trials in which pairs engaged in huddling. (PDF) [file pone.0313172.s003.pdf]
